# The Oxidative Stress Agent Hypochlorite Stimulates c-di-GMP Synthesis and Biofilm Formation in *Pseudomonas aeruginosa*

**DOI:** 10.3389/fmicb.2017.02311

**Published:** 2017-11-22

**Authors:** Nikola Strempel, Michael Nusser, Anke Neidig, Gerald Brenner-Weiss, Joerg Overhage

**Affiliations:** ^1^Institute of Functional Interfaces, Karlsruhe Institute of Technology, Karlsruhe, Germany; ^2^Department of Health Sciences, Carleton University, Ottawa, ON, Canada

**Keywords:** *Pseudomonas aeruginosa*, bacterial stress response, biofilm formation, c-di-GMP, oxidative stress, pathogen

## Abstract

The opportunistic human pathogen *Pseudomonas aeruginosa* is able to survive under a variety of often harmful environmental conditions due to a multitude of intrinsic and adaptive resistance mechanisms, including biofilm formation as one important survival strategy. Here, we investigated the adaptation of *P. aeruginosa* PAO1 to hypochlorite (HClO), a phagocyte-derived host defense compound and frequently used disinfectant. In static biofilm assays, we observed a significant enhancement in initial cell attachment in the presence of sublethal HClO concentrations. Subsequent LC-MS analyses revealed a strong increase in cyclic-di-GMP (c-di-GMP) levels suggesting a key role of this second messenger in HClO-induced biofilm development. Using DNA microarrays, we identified a 26-fold upregulation of ORF PA3177 coding for a putative diguanylate cyclase (DGC), which catalyzes the synthesis of the second messenger c-di-GMP – an important regulator of bacterial motility, sessility and persistence. This DGC PA3177 was further characterized in more detail demonstrating its impact on *P. aeruginosa* motility and biofilm formation. In addition, cell culture assays attested a role for PA3177 in the response of *P. aeruginosa* to human phagocytes. Using a subset of different mutants, we were able to show that both Pel and Psl exopolysaccharides are effectors in the PA3177-dependent c-di-GMP network.

## Introduction

*Pseudomonas* *aeruginosa* is a widespread Gram-negative water and soil bacterium, which is in addition one of the most important opportunistic human pathogens causing severe infections in immunocompromised persons (Hancock and Speert, 2000; Stover et al., 2000). Moreover, *P. aeruginosa* is the leading cause of chronic pneumonia in patients suffering from cystic fibrosis (CF) (Rajan and Saiman, 2002). The high pathogenicity of *P. aeruginosa* is linked to its intrinsic resistance to several commonly used antibiotics, the ability to rapidly adapt to varying environmental conditions and the expression of a large arsenal of virulence factors, which facilitate bacterial invasion and the establishment of persistent infections in host organisms (Breidenstein et al., 2011).

Under environmental stress conditions, *P. aeruginosa* is able to form robust biofilms in which surface-associated bacteria are embedded in a self-produced matrix (extrapolymeric substances; EPS) (Costerton et al., 1994; Hall-Stoodley et al., 2004). This switch from a free-swimming motile to a sessile biofilm lifestyle starts with the attachment of bacterial cells to a surface (Hall-Stoodley et al., 2004) and is regulated by a complex regulatory network of different signaling pathways and is associated with overall changes in gene expression leading to an enhanced matrix production and to a loss of motility-related factors (Whiteley et al., 2001; Dötsch et al., 2012). Among others, nucleotide second messengers such as cyclic di-GMP (c-di-GMP), cell-density dependent quorum sensing, sigma factors and two-component regulatory systems play an important role in biofilm formation (Mikkelsen et al., 2011; Fazli et al., 2014). Living within such a biofilm provides bacteria protection against a variety of different stressors like toxic substances, antibiotics or the host immune system and is therefore considered as an adaptive resistance mechanism (Hall-Stoodley et al., 2004; Breidenstein et al., 2011).

During acute and chronic infections, bacteria are confronted with a variety of host defense mechanisms designed to kill and eliminate invading pathogens from the host organism. These mechanisms include the secretion of compounds with direct antimicrobial or immunomodulatory activities or the incorporation and subsequent clearance of bacteria by phagocytic cells (Williams et al., 2010; Gellatly and Hancock, 2013). With respect to *P. aeruginosa* lung infections, main actors in the fight against this pathogen are epithelial cells and immune cells belonging to the innate immune system, such as phagocytic neutrophils or activated alveolar macrophages (Gellatly and Hancock, 2013; Lovewell et al., 2014). One major killing agent of mammalian phagocytes and, at the same time a frequently used disinfectant, is the potent oxidant hypochlorite (HClO) (Hawkins and Davies, 1998). HClO is generated from hydrogen peroxide (H_2_O_2_) and chloride ions (Cl^-^) in a chemical reaction which is catalyzed by the enzyme myeloperoxidase (MPO) and reacts with a large number of biomolecules, including proteins, lipids and DNA and thus rapidly induces bacterial cell death (Albrich et al., 1981; Rosen et al., 1990; Klebanoff, 2005; Gray et al., 2013a).

While several studies investigated the bacterial stress responses to the less reactive oxidant H_2_O_2_ (Palma et al., 2004; Chang et al., 2005; Salunkhe et al., 2005; Choi et al., 2007; Small et al., 2007a; Heo et al., 2010; Lan et al., 2010; Goldova et al., 2011; Hare et al., 2011; Romsang et al., 2013; Deng et al., 2014) information on the immediate response of *P. aeruginosa* to HClO as a component of the human first line of defense, its intracellular sensing and its implication on the adaptation of *P. aeruginosa* to a hostile environment still remains elusive. Earlier studies have shown that HClO influences energy, iron and polyphosphate metabolism in *P. aeruginosa* (Small et al., 2007a,b; Groitl et al., 2017), however, the impact of HClO on *P. aeruginosa* biofilm initiation and development has not been investigated so far.

In the present study, we show that sublethal HClO concentrations strongly induce early steps of biofilm formation in *P. aeruginosa* PAO1 by increasing intracellular levels of the second messenger c-di-GMP. Further analyses identified the diguanylate cyclase (DGC) PA3177 as part of the HClO-induced stress response and we demonstrate its involvement in motility, biofilm formation and interaction of *P. aeruginosa* with THP-1 macrophages.

## Materials and Methods

### Organisms, Plasmids, Primers, and Growth Conditions

A list of bacterial strains and plasmids used in this study is provided in **Table 1**. *P. aeruginosa* PAO1 was either grown in complex Luria Bertani (LB) Miller broth (Carl Roth, Karlsruhe, Germany) or in BM2 minimal medium [62 mM potassium phosphate buffer, pH 7.0, 7 mM (NH_4_)_2_SO_2_, 2 mM MgSO_4_, 10 μM FeSO_4_, 0.4% (w/v) Glucose] at 37°C and 170 rpm. In BM2-swarm medium, 7 mM (NH_4_)_2_SO_4_ was substituted for 0.5% (w/v) casamino acids. *Escherichia coli* strains DH5α and BL21(DE3) were cultivated in LB medium at 37°C. For overexpression experiments, the arabinose-inducible expression vector pJN105 and the IPTG-inducible vector pET23a were used. Antibiotics ampicillin (100 μg/ml) and gentamicin (10 μg/ml for *E. coli*; 30 μg/ml for *P. aeruginosa*) were added for plasmid selection and/or maintenance.

**Table 1:** Plasmids and bacterial strains used in this study.

	Description	Reference
**Plasmids**		
pJN105	Broad-host-range expression vector derived from pBBR1MCS-5, contains araC-p_BAD_, L-arabinose-inducible, Gm^R^	Newman and Fuqua, 1999
pJN3177	Overexpression of *P. aeruginosa* PA3177; arabinose-inducible, Gm^R^	This study
pJN2133	Overexpression of *P. aeruginosa* PA2133; arabinose-inducible, Gm^R^	Hickman et al., 2005
pET23a	*E. coli* expression vector, pBR322 origin, N-terminal T7-Tag, C-terminal His-Tag, T7 promoter, Amp^R^	Novagen, Merck Millipore
pET3177	Vector for heterologous overexpression of PA3177 in *E. coli* BL21; Amp^R^	This study
***P. aeruginosa* strains**		
PAO1	Wildtype strain	Stover et al., 2000
PAO1-PA3177Ω	PAO1 transposon insertion mutant, ID: PAO1_lux_66:D11, PA3177 knock-out, Tc^R^	Lewenza et al., 2005
PAO1-Δ*pelF*	PAO1 deletion mutant, *pelF* (PA3059) knock-out	Ghafoor et al., 2011
PAO1-Δ*pslA*	PAO1 deletion mutant, *pslA* (PA2231) knock-out	Ghafoor et al., 2011
PAO1-Δ*pelF*Δ*pslA*	PAO1 deletion double mutant, *pelF* and *pslA* knock-out	Ghafoor et al., 2011
PAO1-*flgK*Ω	PAO1 transposon insertion mutant, ID:PAO1_lux_24:H1, *flgK*(PA1086) knock-out, Tc^R^	Lewenza et al., 2005
PAO1-pJN105	Vector control strain	This study
PAO1-pJN3177	Overexpression of PA3177 in strain PAO1	This study
PAO1-pJN2133	Overexpression of PA2133 in strain PAO1	This study
PAO1-pJN3177-*gfp*	Overexpression of a PA3177-GFP fusion protein in strain PAO1	This study
PAO1-Δ*pelF*-pJN3177	Overexpression of PA3177 in strain PAO1-Δ*pelF*	This study
PAO1-Δ*pslA*-pJN3177	Overexpression of PA3177 in strain PAO1-Δ*pslA*	This study
PAO1-Δ*pelF* Δ*pslA*-pJN3177	Overexpression of PA3177 in strain PAO1-Δ*pelF*Δ*pslA*	This study
PAO1-*flgK*Ω-pJN3177	Overexpression of PA3177 in strain PAO1-*flgK*Ω	This study
***E. coli* strains**		
DH5α	Cloning strain	Invitrogen
BL21 (DE3)	Expression strain	Stratagene
BL21-pET23a	Vector control strain	This study
BL21-pET3177	Heterologous expression of PA3177 in strain BL21(DE3)	This study

*Tc^*R*^, tetracycline resistance; Gm^*R*^, gentamicin resistance; Amp^*R*^, ampicillin resistance.*

Hypochlorite was added in the form of aqueous sodium hypochlorite (NaClO) or calcium hypochlorite [Ca(ClO)_2_] solutions as mentioned within the text. For effects caused by both, NaClO and Ca(ClO)_2_ treatment, the general term HClO was used within the manuscript.

Free chlorine concentrations of HClO solutions (VWR, Darmstadt, Germany) were determined prior to each assay using DPD Free Chlorine Powder Pillows (VWR, Darmstadt, Germany) according to the manufacturer’s instructions.

The sequences of DNA primers (Eurofins MWG Operon, Germany) used in these studies are shown in Supplementary Tables S1 and S2.

### Construction of Recombinant *P. aeruginosa* and *E. coli* Strains

Cloning procedures were performed with *E. coli* strain DH5α, proof-reading Platinum^®^ *pfx* DNA polymerase and restriction enzymes from Thermo Fisher Scientific (St. Leon-Rot, Germany) according to standard protocols provided by the manufacturers. Primer sequences for subsequent cloning experiments are shown in Supplementary Table S1.

For overexpression in *P. aeruginosa*, ORF PA3177 was amplified from PAO1 genomic DNA using a XbaI restriction site flanked forward primer and a SacI site flanked reverse primer and the fragment was cloned into the arabinose-inducible broad host range vector pJN105 (Newman and Fuqua, 1999), resulting in pJN3177.

For overexpression in *E. coli* BL21 (DE3), ORF PA3177 was amplified from PAO1 genomic DNA using a BamHI restriction site flanked forward primer and a XhoI site flanked reverse primer followed by in-frame insertion into pET-23a(+) (Novagen, Merck, Darmstadt, Germany). The resulting plasmid pET3177 and the vector control pET23a were then transferred into *E. coli* strain BL21 (DE3) by heat shock transformation (Hanahan, 1983). *E. coli* was chosen for the heterologous expression of PA3177 due to its fast growth to high cell densities and the availability of highly efficient and tightly regulated gene expression systems.

### Susceptibility Tests in Microtiter Plates

Minimal inhibitory concentrations (MIC) of different antimicrobials for planktonic *P. aeruginosa* cells were assayed by a standard broth microdilution method in BM2 medium as described previously (Wiegand et al., 2008). Additionally, growth of *P. aeruginosa* PAO1 during 24 h in the presence of different NaClO concentrations was monitored using a microplate reader (TECAN, Männerdorf, Switzerland) under shaking conditions at 37°C.

### Attachment Assay in Microtiter Plates

Attachment assays in 96-well polystyrene microtiter plates (Nunc, Thermo Fisher Scientific, St. Leon-Rot, Germany) were performed as previously described (Overhage et al., 2008) with the following minor modifications. *P. aeruginosa* PAO1 overnight cultures were washed and resuspended in BM2 medium, diluted to an optical density (OD_600_) of 0.2 and transferred to microtiter plates. In case of antimicrobial treatment, respective agents were added at indicated sub-MIC concentrations. Attachment of *P. aeruginosa* overexpression strains PAO1-pJN105, PAO1-pJN3177 or PAO1-pJN2133, was evaluated in BM2 medium supplemented with 0.1% (w/v) L-arabinose (Sigma–Aldrich, Seelze, Germany) to induce recombinant gene expression. Microtiter plates were incubated for 2 h at 37°C without shaking followed by the quantification of biofilm biomass by crystal violet staining as previously described (Merritt et al., 2005). Experiments were performed at least in triplicate with multiple wells per condition. Statistical significance was verified by the non-parametric Mann–Whitney test.

### Attachment on Microscope Slides and Fluorescence Microscopy

Attachment to a surface or interface is the initial step in the formation of biofilms. Attachment of *P. aeruginosa* PAO1 on glass microscope slides in the presence or the absence of NaClO (2 μg/ml) was analyzed as described previously (Bernard et al., 2009). The starting OD_600_ was 0.2 and the slides were incubated for 2 h at 37°C without shaking. Following fixation with 3% (v/v) formaldehyde, the attached bacteria were stained with the DNA intercalating fluorescent dye SYTO9 (Life Technologies, Darmstadt, Germany) according to the manufacturer’s instructions. Fluorescence microscopy was carried out using an Axioplan 2 imaging system (Carl Zeiss, Oberkochem, Germany) with appropriate filter sets at 100 x magnification. Experiments were performed in duplicate and repeated three times. For biomass quantification, the surface coverage of four different squares with a side length of 200 μm was calculated for each slide using *ImageJ* software. All data were statistically analyzed by the non-parametric Mann–Whitney test.

### Nucleotide Extraction and c-di-GMP Quantification by LC-MS

Intracellular c-di-GMP levels in *P. aeruginosa* PAO1 and in *E. coli* BL21 were quantified using a liquid chromatography coupled tandem mass spectrometry (LC-MS) method described by Spangler et al. (2010). *P. aeruginosa* PAO1 overnight cultures were washed and resuspended in BM2 medium. The OD_600_ was adjusted to 1.0 in order to increase the cell mass required for the subsequent extraction procedure, and the cells were incubated in small petri dishes with sublethal concentrations of NaClO (8 μg/ml, due to increased starting cell numbers) or without NaClO for 1 h at 37°C. In case of experiments with PA3177-overexpressing *E. coli* strain BL21-pET3177 and vector control BL21-pET23a, bacterial overnight cultures were diluted 1:100 in LB containing ampicillin (100 μg/ml) and grown until mid-log phase at 37°C under shaking conditions. IPTG was added at a final concentration of 0.4 mM and bacteria were incubated for 5 h at 37°C and 170 rpm. Harvesting and nucleotide extractions were performed as described previously (Spangler et al., 2010; Strehmel et al., 2015) using 200 ng cXMP (Biolog, Bremen, Germany) as an internal standard. Extractions were performed in triplicate and assays were repeated at least with two independent bacterial cultures. Total protein contents of the samples were determined by the Pierce BCA Protein Assay Kit (Thermo Fisher Scientific, St. Leon-Rot, Germany) according to the manufacturer’s instructions. LC-MS measurements were performed as described by Strehmel et al. (2015). Concentrations of c-di-GMP were normalized against total protein contents of respective cultures. Statistical significance was analyzed by the non-parametric Mann–Whitney test.

### Gene Expression Analyses

For microarray analysis, overnight cultures of *P. aeruginosa* PAO1 were washed and resuspended in BM2 medium. After dilution to an OD_600_ of 0.2, 10 ml of the suspensions was transferred to a small petri dish (5 cm diameter; Sarstedt, Nuembrecht, Germany) and NaClO was added at a final concentration of 2 μg/ml. Untreated samples served as negative controls. Bacteria were incubated for 1 h at 37°C, mimicking growth during static attachment assays in 96-well microtiter plates in order to increase the cell mass required for subsequent RNA extractions and microarray analysis. RNA extraction, cDNA synthesis and processing was performed as described previously (Strempel et al., 2013). Samples from three independent experiments per condition each with a minimum of two technical replicates were pooled to detect significantly up- or down-regulated genes. Each sample was hybridized to two microarray *Affymetrix* Pae_G1a GeneChips (*n* = 4). Only genes which were more than 3-fold dysregulated in NaClO-treated bacteria were included in further analyses (Student *t*-test *p*-value ≤ 0.05). Complete microarray data is deposited in the ArrayExpress database under accession number E-MTAB-2876. These results were verified using qRT-PCR as described in the following on three additional, independent experiments.

For quantitative realtime-PCR (qRT-PCR), *P. aeruginosa* PAO1 overnight cultures were washed and diluted with BM2 medium to an OD_600_ of 0.2 followed by incubation with NaClO, Ca(ClO)_2_, NH_2_Cl, paraquat and H_2_O_2_ at indicated concentrations for 1 h at 37°C. For each condition, three independent experiments each with a minimum of two technical replicates were conducted. Untreated bacteria served as negative controls. RNA isolation, cDNA synthesis and subsequent qRT-PCR experiments were carried out as described previously (Strempel et al., 2013). Obtained Ct values were normalized to expression of housekeeping genes *rpoD* and *fabD* as described elsewhere (Schmidberger et al., 2013).

### Motility Assays

Motility assays were performed as described elsewhere (Strehmel et al., 2015). Overnight cultures of strains PAO1-pJN3177 and PAO1-pJN105 were diluted 1:50 in LB medium containing 30 μg/ml gentamicin and 0.1% (w/v) arabinose and incubated for 5 h at 37°C and 170 rpm prior to inoculation of agar plates. LB or BM2 agar plates were also supplemented with 0.1% (w/v) arabinose and 30 μg/ml gentamicin. Assays were performed in triplicate and mean values of five plates per experiment were calculated. Statistical significance of the obtained results was examined by the non-parametric Mann–Whitney test.

### Phagocytosis Assays with Human THP-1 Macrophages

Gene expression in phagocytized bacteria was analyzed using qRT-PCR following interaction with THP-1 human macrophage-like cells and *P. aeruginosa* PAO1. THP-1 cells (German Collection of Microorganisms and Cell Cultures – DSMZ, Braunschweig, Germany) were routinely cultured in complete RPMI-1640medium containing 10% (v/v) heat-inactivated FBS and 2 mM L-glutamine (Sigma–Aldrich, Hamburg, Germany) at 37°C and 5% CO_2_. To start the experiment, cells were seeded out in 6-well microtiter plates at a density of 5 × 10^6^cells per well. Differentiation into adherent macrophage-like cells was stimulated by the addition of 5 ng/ml PMA (Sigma–Aldrich, Hamburg, Germany) for 48 h as described previously (Park et al., 2007). *P. aeruginosa* PAO1 overnight cultures grown in BM2 were washed twice in complete RPMI medium and added to the THP-1 macrophages at a multiplicity of infection of 100. Interaction assays were carried out for 1h at 37°C. THP-1 cells without bacteria and *P. aeruginosa* incubated without THP-1 macrophages served as controls. After phagocytosis, supernatants which contained residual planktonic bacteria were removed from the wells. After a washing step with PBS, complete RPMI medium supplemented with the antibiotic gentamicin at a final concentration of 400 μg/ml was added to the THP-1 monolayer and remaining extracellular bacteria were killed during a 30 min incubation step at 37°C as described previously (Neidig et al., 2013). Subsequently, the THP-1 cells were rinsed with complete RPMI medium and incubated with a 1:1 (v/v) mixture of *RNAprotect Bacteria Reagent* and complete RPMI medium for 5 min. During this incubation step, adherent THP-1 cells detached from the bottom of the 6-well plate and could be easily harvested by centrifugation (10 min, 5,000 × *g*, RT). All control samples were also treated with a 1:1 (v/v) mixture of *RNAprotect Bacteria Reagent* and complete RPMI medium prior to RNA isolation. Supernatants were discarded and cell pellets were stored at -80°C until needed. Total RNA was extracted using the *RNeasy Mini Kit* in combination with *QIAshredder* columns (Qiagen, Hilden, Germany) according to the manufacturer’s protocol for total RNA isolation from animal cells. The obtained RNA was directly used for cDNA synthesis and subsequent qRT-PCR.

*In vitro* phagocytosis of PAO1 cultures by THP-1 was performed as described previously (Yeung et al., 2014) using mid-log bacterial cells and THP-1 at an MOI of 10. Three independent experiments with triplicates in each experiment were performed for each bacterial strain.

## Results

### HClO Induces Biofilm Formation in *P. aeruginosa* PAO1

In order to determine appropriate NaClO concentrations for subsequent assays, the susceptibility of *P. aeruginosa* PAO1 toward NaClO was analyzed by MIC determination in BM2 minimal medium resulting in MIC values of 4 μg/ml free chlorine. This value was confirmed by monitoring bacterial growth during 24 h in the presence of different concentrations of NaClO (Supplementary Figure S1). Thus, for subsequent experiments sublethal concentrations of 2 μg/ml free chlorine were chosen.

**FIGURE 1:**
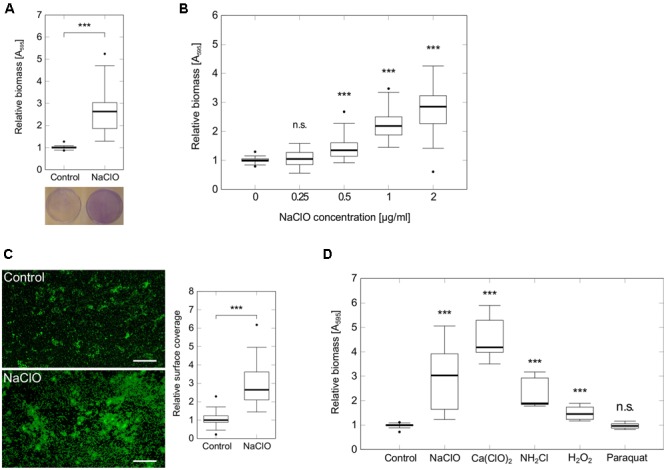
Attachment of *P. aeruginosa* PAO1 in the presence of different oxidants. Attachment of *P. aeruginosa* PAO1 during 2 h incubation in the presence of sublethal NaClO concentrations **(A–C)** or half-MIC concentrations of oxidants NaClO (2 μg/ml), Ca(ClO)_2_ (2 μg/ml), NH_2_Cl (equivalent to 2 μg/ml NaClO), H_2_O_2_ (50 μg/ml) and paraquat (1 μg/ml) **(D)**. **(A,B,D)** Attachment was assayed in 96-well microtiter plates by crystal violet staining and subsequent measurement of A_595_. Obtained values for treated samples were normalized against A_595_ of untreated controls (=relative biomass). Experiments were performed at least in triplicate, each with multiple wells per condition (*n* ≥ 18). **(C)** *P. aeruginosa* PAO1 was incubated with sub-MIC concentrations of NaClO (2 μg/ml) in BM2 for 2 h in 50 ml reaction tubes containing glass microscope slides. Adhered bacteria were stained with the DNA-intercalating dye SYTO9 prior to visualization by fluorescence microscopy at 100× magnification (scale bar: 100 μm). Average surface coverages from 36 pictures per condition were calculated using *ImageJ*. Experiments were performed in triplicate. For all experiments, statistical significance was evaluated by the Mann-Whitney test (^∗∗∗^*p* ≤ 0.001, n.s., not significant). Boxes include median (thick horizontal line), 25th and 75th percentiles. Dots indicate extreme values considered as outliers.

Since adhesion of bacterial cells to a surface is among the first steps in the development of biofilms (Hall-Stoodley et al., 2004), we performed static attachment assays in 96-well microtiter plates to show a significant 2.8-fold increase in initial adhesion of *P. aeruginosa* PAO1 during 2 h exposure to 2 μg/ml NaClO (**Figure 1A**). Additional tests indicated a dose-dependent induction of attachment starting at concentrations as low as 0.5 μg/ml NaClO (**Figure 1B**). To verify these findings, *P. aeruginosa* attachment was also evaluated on glass microscope slides using fluorescence microscopy to show a similar 2.6-fold increase in the amount of attached bacteria in the presence of NaClO (**Figure 1C**).

In order to investigate whether this enhanced attachment was part of the general response to oxidative stress in *P. aeruginosa*, bacterial adhesion was monitored under different oxidative stress conditions. Evaluated substances included H_2_O_2_, calcium hypochlorite [Ca(ClO)_2_], paraquat and monochloramine (NH_2_Cl), a downstream reaction product of HClO. In each case, half of the determined MIC was used for the experiment [H_2_O_2_: 50 μg/ml, Ca(ClO)_2_: 2 μg/ml, Paraquat: 1 μg/ml]. NH_2_Cl and NaClO were applied at a molecular ratio of 1:1. Thus, final concentrations of NH_2_Cl were equivalent to concentrations resulting from the reaction of 2 μg/ml NaClO with amines. In addition to NaClO, the strongest induction of attachment was found in response to Ca(ClO)_2_ (+4.2-fold) and NH_2_Cl (+1.9-fold). For H_2_O_2_, there was still a slight increase in attachment detectable (+1.5-fold), whereas cells incubated with paraquat did not show any difference in biomass compared to untreated controls (**Figure 1D**) indicating that attachment is not induced by oxidative stress in general.

### The Second Messenger c-di-GMP Is Involved in the NaClO-Induced Biofilm Formation

**FIGURE 2:**
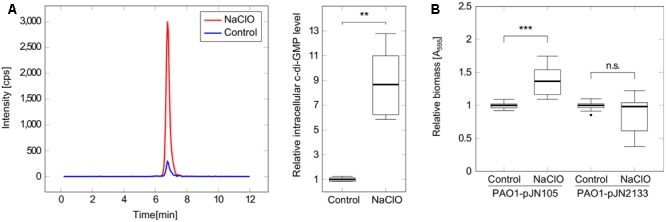
Implication of c-di-GMP in the response of *P. aeruginosa* PAO1 to NaClO. **(A)** *P. aeruginosa* PAO1 was incubated with NaClO (8 μg/ml, OD_600_ = 1.0, BM2) for 1h followed by nucleotide extraction and quantification of intracellular c-di-GMP by LC-MS. Levels of c-di-GMP were normalized to the total protein content of the respective sample and compared to c-di-GMP levels in untreated controls (*n* = 6). The chromatogram shows c-di-GMP peaks of one representative measurement. **(B)** Attachment of *P. aeruginosa* in response to NaClO during overexpression of the c-di-GMP-degrading PDE PA2133 was evaluated by crystal violet staining. PAO1-pJN2133 and the vector control strain PAO1-pJN105 were incubated in 96-well microtiter plates either in the presence or in the absence of NaClO (2 μg/ml) for 2 h at 37°C followed by the quantification of biofilm biomass. BM2 was supplemented with 0.1% (w/v) arabinose to induce recombinant gene expression. Experiments were repeated four times, each with six wells per strain and condition (*n* = 24). Absorbance at 595nm (A_595_) was determined and obtained values for NaClO-treated samples were normalized against the A_595_ of respective untreated controls (=relative biomass). Statistical significance was evaluated by the Mann–Whitney test (^∗∗∗^*p* ≤ 0.001, ^∗∗^*p* ≤ 0.01, n.s., not significant). Boxes include median (thick horizontal line), 25th and 75th percentiles. Dots indicate extreme values considered as outliers.

*Pseudomonas aeruginosa* biofilm development is a complex adaptation which is regulated by a multitude of different signaling pathways including the second messenger c-di-GMP (Kalia et al., 2013; Fazli et al., 2014). In order to evaluate the involvement of c-di-GMP in the observed NaClO phenotype, intracellular c-di-GMP levels were quantified using LC-MS to show a significant increase in c-di-GMP levels by 8.7-fold in bacteria treated with NaClO (**Figure 2A**). With respect to absolute c-di-GMP levels, average concentrations of 201 pmol and 25 pmol c-di-GMP per mg protein in NaClO-treated and control cultures, respectively, were measured.

In order to test whether these increased c-di-GMP levels were an important factor for the NaClO-triggered initiation of biofilm formation, additional assays were performed with strain *P. aeruginosa* PAO1-pJN2133, which overexpresses the phosphodiesterase (PDE) PA2133 in the presence of 0.1% (w/v) arabinose leading to an enhanced and continuous c-di-GMP degradation and subsequently low intracellular c-di-GMP levels within the cell. Crystal violet staining after 2 h revealed a significant 1.4-fold increase in adhered biomass of PAO1-pJN105 in the presence of NaClO compared to the untreated control. In contrast, attachment was not affected in NaClO-treated cells of PAO1-pJN2133 in comparison to untreated bacteria (**Figure 2B**) suggesting an important role for c-di-GMP in NaClO-induced biofilm formation of *P. aeruginosa*.

### Expression of the Putative DGC PA3177 Is Induced by HClO

Intracellular c-di-GMP levels are regulated by the activity of DGCs which catalyze c-di-GMP synthesis from two GTP molecules and c-di-GMP-degrading PDEs. DGCs are characterized by a conserved GGDEF domain, whereas PDEs harbor either an EAL or a HD-GYP motif (Hengge, 2009; Kalia et al., 2013). To investigate which DGCs or PDEs are responsible for the increase in c-di-GMP upon NaClO treatment, global gene expression analyses using DNA microarrays were performed and gene expression levels in response to NaClO (2 μg/ml, 1 h) were compared to those of untreated PAO1 cells. Out of the 33 genes which possess a DGC consensus sequence in *P. aeruginosa* (Kulasakara et al., 2006; Winsor et al., 2011), only ORF PA3177 showed statistically significant changes in gene expression levels and an upregulation by 26.2-fold in response to NaClO. Genes coding for PDEs were not affected by the oxidant (ArrayExpress E-MTAB-2876). The increase in PA3177 gene expression in response to NaClO was confirmed by qRT-PCR (**Table 2**). In addition, qRT-PCR experiments with the oxidants H_2_O_2_, paraquat, Ca(ClO)_2_ and NH_2_Cl indicated that the induction of PA3177 was not part of the general response of *P. aeruginosa* to oxidative stress. A strong increase in PA3177 expression was only found upon treatment with Ca(ClO)_2_, NH_2_Cl and NaClO. In contrast, in response to paraquat, PA3177 levels were only slightly enhanced and H_2_O_2_ did not affect PA3177 gene expression at all (**Table 2**).

**Table 2:** PA3177 gene expression in response to different oxidants^a^.

Compound	Applied concentration	Relative gene expression^c^
NaClO	2 μg/ml free chlorine^b^	13.7 ± 0.9
Ca(ClO)_2_	2 μg/ml free chlorine	86.0 ± 4.0
NH_2_Cl	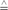 2 μg/ml free chlorine	28.6 ± 4.9
H_2_O_2_	50 μg/ml (1.47 mM)	1.2 ± 0.1
Paraquat	1 μg/ml (3.88 mM)	2.2 ± 0.2

*^a^*P. aeruginosa* PAO1 was incubated with different oxidants at indicated half-MIC concentrations in BM2 minimal medium for 1 h at 37°C followed by RNA isolation and qRT-PCR. ^b^Free chlorine concentrations are shown in μg/ml as determined by the respective test kit. Since free chlorine is defined as a mixture of HClO, ClO^-^ and Cl_*2*_ and therefore possesses a variable molecular mass, corresponding millimolar concentrations were not calculated. ^c^Mean averages and standard deviations of three independent experiments, each analyzed at least in duplicate (*n* ≥ 6). Ct values were normalized against expression of housekeeping genes *rpoD* and *fabD* and relative PA3177 expression in treated cells compared to untreated controls was calculated.*

### Characterization of the DGC PA3177

According to the *Pseudomonas* Genome Database (Winsor et al., 2011), the gene product of ORF PA3177 is a hypothetical cytoplasmic protein with a predicted molecular weight of 34.4 kDa containing a GGDEF domain (here: GGEEF). However, previous studies failed to confirm a c-di-GMP-generating enzyme activity of the PA3177 homologue in *P. aeruginosa* PA14 and did not show a consistent impact of PA3177 on biofilm formation (Kulasakara et al., 2006; Merritt et al., 2010).

**FIGURE 3:**
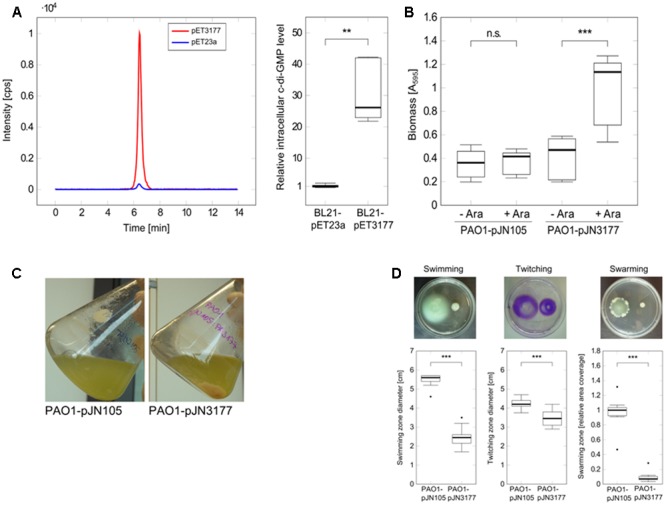
Characterization of the DGC PA3177. **(A)** *E. coli* BL21-pET3177 and the vector control strain BL21-pET23a were grown in LB broth supplemented with 0.4 mM IPTG for 5 h at 37°C followed by nucleotide extraction and quantification of intracellular c-di-GMP by LC-MS. Levels of c-di-GMP were normalized to the total protein content of the respective sample and compared to c-di-GMP levels in empty vector control strain BL21-pET23a (*n* = 6). The chromatogram shows c-di-GMP peaks of one representative measurement. **(B)** Attachment of *P. aeruginosa* during PA3177 overexpression was evaluated by crystal violet staining. PAO1-pJN3177 and empty vector control strain PAO1-pJN105 were incubated in 96-well microtiter plates for 2 h at 37°C followed by biomass quantification. For recombinant gene expression, BM2 was supplemented with 0.1% (w/v) arabinose (+Ara). Cultures without arabinose (–Ara) served as negative controls. Experiments were carried out in triplicate, each with six wells per strain and condition (*n* = 18). **(C)** Planktonic growth during 5 h at 37°C. **(D)** Overnight cultures of PA3177-overexpressing strain *P. aeruginosa* PA01-pJN3177 and vector control PAO1-pJN105 were diluted in LB broth supplemented with 0.1% (w/v) arabinose and grown for 5 h at 37°C followed by evaluation of swimming, swarming and twitching motility. Assays were carried out with three independent bacterial cultures and at least four agar plates per experiment (*n* ≥ 12). In all experiments statistical significance was evaluated by the Mann-Whitney test (^∗∗∗^*p* ≤ 0.001, ^∗∗^*p* ≤ 0.01, n.s., not significant). Boxes include median (thick horizontal line), 25th and 75th percentiles. Dots indicate extreme values considered as outliers.

To investigate the DGC activity of *P. aeruginosa* PAO1 PA3177, the enzyme was heterologously expressed for 5 h in *E. coli* BL21-pET3177 to obtain a high protein concentration, which was confirmed by SDS-PAGE and MALDI-ToF mass spectrometry analysis. Nucleotides were extracted from BL21-pET3177 and subsequent quantification of intracellular c-di-GMP levels by LC-MS revealed a 26.1-fold increase in c-di-GMP synthesis upon overexpression of PA3177 compared to BL21-pET23a (**Figure 3A**), which confirmed its DGC activity.

To further characterize this DGC, the PA3177 overexpressing strain PAO1-pJN3177 was constructed in which PA3177 expression was under the control of an arabinose-inducible promoter. After 2 h of PA3177 expression, cultures of PAO1-pJN3177 showed a 2.4-fold enhancement in biofilm biomass in 96-well microtiter plates in comparison to vector control PAO1-pJN105 or uninduced PAO1-pJNPA3177 cultures (**Figure 3B**).

Since motility is also strongly regulated by c-di-GMP (Hengge, 2009), the main motility types of *P. aeruginosa* – swimming in aqueous environments, twitching on solid surfaces and swarming on semisolid surfaces – were evaluated during PA3177 overexpression. To this aim, overnight cultures of *P. aeruginosa* PAO1-pJN3177 and control strain PAO1-pJN105 were diluted 1:50 in LB broth supplemented with 0.1% (w/v) arabinose and grown for 5 h at 37°C. Of note, at this time point PAO1-pJN3177 formed already clearly visible cell aggregates in the flask indicating DGC activity (**Figure 3C**). In agreement with other studies which identified several DGCs as negative regulators of *P. aeruginosa* motility (Aldridge et al., 2003; Merritt et al., 2007), a 61% decrease in swimming ability was observed upon induction of PA3177 expression. Similar results were obtained for twitching motility after 72 h at 37°C (18% decrease) and swarming motility after 21 h at 37°C (99% decrease) in response to PA3177 overexpression (**Figure 3D**).

### Role of Pel and Psl exopolysaccharides

**FIGURE 4:**
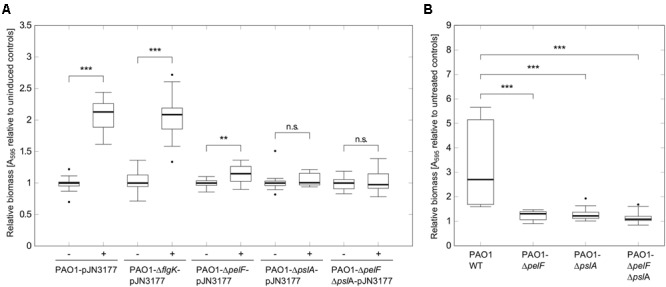
Attachment assays with PAO1 mutants. **(A)** Attachment of *P. aeruginosa* PAO1 wildtype (WT) or PAO1 mutant strains during overexpression of PA3177 was evaluated by crystal violet staining. Bacteria were incubated in 96-well microtiter plates for 2 h at 37°C prior to biomass quantification. For recombinant gene expression, L was supplemented with 0.1% (w/v) arabinose (+). Cultures without arabinose (-) served as negative controls. **(B)** Attachment of *P. aeruginosa* PAO1 WT or PAO1 deletion mutants during NaClO treatment (2 μg/ml in BM2, 2 h) was evaluated by crystal violet staining. All experiments were carried out in triplicate, each with six wells per strain and condition (*n* = 18). Statistical significance was evaluated by the Mann–Whitney test (^∗∗∗^*p* ≤ 0.001, ^∗∗^*p* ≤ 0.01, n.s., not significant). Boxes include median (thick horizontal line), 25th and 75th percentiles. Dots indicate extreme values considered as outliers.

Several DGCs have been shown to induce attachment and biofilm formation by influencing flagella movement or stimulating extracellular matrix production (Klebensberger et al., 2009; Boehm et al., 2010; Malone et al., 2010; Petrova et al., 2014). To investigate in which way PA3177 modulates biofilm formation, plasmid pJN3177was transferred to a set of different *Pseudomonas aeruginosa* PAO1 mutants and attachment of respective strains was quantified after PA3177 induction. In addition to a *flgK* mutant with a defect in flagellum synthesis, mutants with defects in the Pel or Psl exopolysaccharide biosynthesis clusters were tested, since Pel and Psl have been identified to be important components for attachment and early biofilm formation in *P. aeruginosa* PAO1 (Evans and Linker, 1973; Friedman and Kolter, 2004; Jackson et al., 2004; Overhage et al., 2005). As shown in **Figure 4A**, a disrupted *pslA* gene or a simultaneous knock-out of Pel and Psl in the double mutant PAO1-Δ*pelF*Δ*pslA*-pJN3177 completely abolished the biofilm-inducing effect of PA3177. Furthermore, the single mutant PAO1-*pelF*-pJN3177 showed only a marginal 1.1-fold increase in attachment when PA3177 was overexpressed. In contrast, in the absence of *flgK*, overexpression of PA3177 in strain PAO1-*flgK*Ω-pJN3177 led to a 2.1-fold increase in attachment which was comparable to PAO1-pJN3177 cells (**Figure 4A**). In addition, we analyzed gene expression of *pelA* and *pslA* in PAO1-pJN3177and PAO1-pJN105 using qRT-PCR. Since the microarray data did not reveal any upregulation in gene expression after 1 h of incubation and to ensure appropriate expression levels of PA3177 in PAO1-pJN3177, we determined *pelA* and *pslA* gene expression after 4 h of PA3177 expression. In comparison to the vector control strain PAO1-pJN105, we obtained an enhanced expression of *pelA* and *pslA* by 2.6 ± 0.8-and 7.9 ± 1.0-fold, respectively, after 4 h of PA3177 over-expression.

Next, we investigated whether Pel and Psl were implicated in the enhanced biofilm initiation in response to NaClO, i.e., under conditions in which the expression of PA3177 was naturally induced in PAO1 wildtype cells. To this aim, attachment of the deletion mutants PAO1-Δ*pelF*, PAO1*-*Δ*pslA* and of the double mutant PAO1-Δ*pelF*Δ*pslA* was analyzed in the presence of 2 μg/ml NaClO. Similar to PA3177 overexpression, NaClO treatment led to a significantly lower induction of attachment in the respective mutant strains in comparison to PAO1 wildtype (**Figure 4B**). Thus, the results demonstrate that both Pel and Psl polysaccharides are involved in the NaClO- and PA3177-dependent stimulation of biofilm formation in *P. aeruginosa* PAO1.

### Identification of Alternative NaClO-Responsive DGCs

**FIGURE 5:**
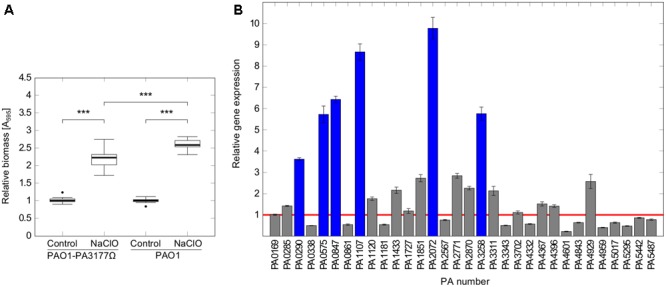
Attachment and DGC expression in PAO1-PA3177Ω in response to NaClO. **(A)** Attachment of *P. aeruginosa* mutant strain PAO1-PA3177Ω and wildtype PAO1 after 2 h incubation with half-MIC concentrations of NaClO (2 μg/ml) compared to untreated controls was assayed in 96-well microtiter plates by crystal violet staining. To show alterations in attachment caused by NaClO, A_595_ of treated samples was normalized to A_595_ of untreated controls for each strain (=relative biomass). Statistical significance was evaluated by the Mann–Whitney test (^∗∗∗^*p* ≤ 0.001, *n* ≥ 12). Boxes include median (thick horizontal line), 25th and 75th percentiles. Dots indicate extreme values considered as outliers. **(B)** *P. aeruginosa* mutant strain PAO1-PA3177Ω was treated with 2 μg/ml NaClO and relative gene expression levels of genes encoding DGCs in comparison to untreated control cultures were analyzed by qRT-PCR. The figure shows mean averages and standard deviations calculated from three experiments, each analyzed in duplicate (*n* = 6). Ct values were normalized against the expression of housekeeping genes *rpoD* and *fabD*. The red line corresponds to a relative gene expression level of 1, which means no change in gene expression. Blue bars indicate relative gene expression values ≥ 3.

A *P. aeruginosa* PA3177 knock-out mutant (PAO1-PA3177Ω) of the *Pseudomonas aeruginosa* PAO1 mini-Tn*5 lux* transposon mutant library (Lewenza et al., 2005) was used in the following experiments. The correct insertion of the transposon cassette was confirmed by colony PCR. Susceptibility tests revealed equal MIC values of 4 μg/ml NaClO for PAO1-PA3177Ω and PAO1 wildtype. Phenotypical characterization of the PAO1-PA3177Ω mutant showed no changes in swimming, swarming or twitching motility as well as the ability to form biofilms compared to PAO1 wildtype (data not shown). Furthermore, subsequent attachment assays in the presence of NaClO demonstrated that a knock-out of PA3177 does not completely impede the biofilm-inducing effect of NaClO, since attachment of the mutant was still significantly enhanced compared to the untreated control (**Figure 5A**). However, this increase in attachment by 2.2-fold was significantly lower than the 2.6-fold increase of PAO1 wildtype.

The *P. aeruginosa* PAO1 genome encodes for at least 33 proteins with the GGDEF consensus sequence of DGCs (Kulasakara et al., 2006). Of note, not all of these proteins contain an intact GGDEF domain, and 16 proteins are hybrid proteins harboring an additional EAL domain being a typical functional domain of c-di-GMP-degrading phosphodiesterases (PDEs) (Kulasakara et al., 2006; Winsor et al., 2011). Most of these hybrid proteins possess either a DGC or a PDE activity, whereas a dual DGC-PDE function has been confirmed for only few enzymes (e.g., PA4601) so far (Phippen et al., 2014). In contrast, the hybrid enzymes PA2567 and PA5017 have been identified as functional PDEs (Rao et al., 2009; Roy et al., 2012). So far, an actual DGC activity has been proposed for at least eleven putative DGCs of *P. aeruginosa* (Kulasakara et al., 2006; Merritt et al., 2007; Klebensberger et al., 2009; Petrova et al., 2014). Therefore, we hypothesized that in case of a PA3177 knock-out, alternative DGCs may adopt functions of PA3177 with respect to NaClO induced biofilm formation.

To test this idea, PAO1-PA3177Ω was incubated in the presence of sub-MIC concentrations of NaClO and expression of all putative DGCs was analyzed by qRT-PCR in order to detect changes in gene expression compared to untreated controls. Indeed, NaClO treatment induced the expression of additional six alternative DGCs in the PAO1-PA3177Ω knock-out mutant by more than three-fold (**Figure 5B**). The strongest upregulation was found for PA2072 (9.8 ± 0.5-fold) and PA1107 (8.7 ± 0.4-fold), followed by PA0847 (6.4 ± 0.2-fold), PA3258 (5.8 ± 0.3-fold), PA0575 (5.7 ± 0.4-fold) and PA0290 (3.6 ± 0.1-fold). Of note, none of the mentioned DGCs were identified differentially regulated in above described microarray analysis of NaClO-treated PAO1 (ArrayExpressE-MTAB-2876). Additional qRT-PCR experiments on different cultures confirmed these results for PA0290, PA0575, PA0847, PA3258. Interestingly, an upregulation of PA1107 (5.5 ± 0.2-fold) and PA2072 (5.4 ± 0.2-fold) was observed in addition to PA3177 (13.7 ± 0.9-fold) in NaClO-treated PAO1 wildtype cells, however, the induction of these two DGCs in PAO1 WT was considerably lower than in the mutant strain PAO1-PA3177Ω. Overall, these results suggest that in addition to PA3177 additional DGCs exhibit an increase in gene expression in response to NaClO in the PAO1 wildtype and the PAO1-PA3177Ω mutant strain, indicating that a knock-out of PA3177 can be counterbalanced by one or more additional DGC activities within the c-di-GMP network of PAO1.

### PA3177 Expression Is Induced in Contact with Phagocytes

HClO is generated in human immune cells during phagocytosis in a chemical reaction of H_2_O_2_ and Cl^-^ which is catalyzed by the MPO enzyme (Klebanoff, 2005; Groitl et al., 2017). Since HClO was able to activate PA3177 gene expression *in vitro*, in the next step, PA3177 expression was examined in *P. aeruginosa* PAO1 ingested by phagocytic cells in order to identify a role of PA3177 during host–pathogen interactions. Phagocytosis experiments were carried out with human THP-1 macrophage-like cells and *P. aeruginosa* PAO1 at a multiplicity of infection of 100. MPO expression in THP-1 macrophages had been previously confirmed by Hachiya et al. (2000). Phagocytic uptake of bacteria by THP-1 macrophages was confirmed by fluorescence microscopy using a *P. aeruginosa* strain which constitutively expressed the green fluorescent protein GFP. As a result, expression of PA3177 was upregulated by 9.5 ± 0.4-fold in phagocytized bacteria compared to PAO1 cells grown without any contact to THP-1 macrophages. However, when we investigated virulence of PAO1-PA3177Ω and PAO1 wildtype during phagocytosis using THP-1 cells and an MOI of 10, we did not observe any statistically significant changes in survival rates after 2 h of incubation (Supplementary Figure S2).

## Discussion

The second messenger c-di-GMP acts as a key regulator of motility and biofilm formation in many bacteria. Although much effort has been put into the elucidation of *P. aeruginosa* c-di-GMP signaling during the last decade, there is still a need for further investigation in order to obtain a complete view of cellular c-di-GMP actions and their implication in *P. aeruginosa* stress response and host-pathogen interactions (Hengge, 2009; Römling et al., 2013). For example, only a few environmental stimuli which influence the synthesis or degradation of c-di-GMP have been identified so far (Hoffman et al., 2005; Klebensberger et al., 2009; Malone et al., 2012; Li et al., 2013; Luo et al., 2015), and knowledge about detailed c-di-GMP effector pathways and connections to other stress-responsive networks is still incomplete (Hengge, 2009; Römling et al., 2013).

In this study, we demonstrate that the phagocytic killing agent and strong disinfectant HClO acts as an activator of c-di-GMP synthesis and subsequent attachment and biofilm formation in *P. aeruginosa*. The hyperattachment phenotype is prevented by overexpression of the c-di-GMP-degrading PDE PA2133 enabling a constant degradation of c-di-GMP in the respective strain *P. aeruginosa* PAO1-pJN2133 (Hickman et al., 2005) during NaClO incubation, which highlights the role of the second messenger in this adaptation process. Further results suggest an involvement of the DGC PA3177 whose expression was strongly stimulated by the HClO-releasing agents NaClO and Ca(ClO)_2_ and NH_2_Cl, which is an abundant secondary reaction product of HClO and nitrogen-containing organic compounds. In contrast, transcriptional levels were not altered or only marginally enhanced in response to the oxidants H_2_O_2_ and paraquat.

Several studies present evidence for the ability of bacteria to increase biofilm formation in response to sublethal concentrations of otherwise highly toxic agents such as antibiotics, detergents and biocides (Bagge et al., 2004; Hoffman et al., 2005; Linares et al., 2006; Klebensberger et al., 2009; Shemesh et al., 2010; Damron et al., 2011; Kaplan, 2011; Malone et al., 2012). However, a regulatory impact on c-di-GMP metabolism in *P. aeruginosa* has only been described for some agents including tobramycin and SDS and both toxic agents promote a sessile mode of growth of *P. aeruginosa* when applied at sublethal concentrations (Hoffman et al., 2005; Klebensberger et al., 2009). So far, the only studies in which sublethal doses of NaClO are linked to an enhanced bacterial biofilm formation deals with *E. coli* (Capita et al., 2014) and *Staphylococcus aureus* MRSA (Buzon-Duran et al., 2017). The authors propose that an increase in cell surface hydrophobicity caused by NaClO is responsible for the observed induction of biofilm formation in *E. coli*. However, a potential implication of c-di-GMP in the observed phenotype was not suggested in the respective study(Capita et al., 2014).

Our results indicate that the DGC PA3177 is specifically induced by HClO but not by oxidative stress in general. With respect to the tested agents, both HClO and NH_2_Cl as well as H_2_O_2_ are able to directly generate oxidative damage of cellular macromolecules and additionally promote the synthesis of further highly reactive oxygen species, e.g., hydroxyl radicals (⋅OH) and singlet oxygen (^1^O_2_) which in turn intensifies the oxidative cell injury (Fang, 2004; Klebanoff, 2005). Similar mechanisms have been reported for the herbicide paraquat whose oxidative potential is predominantly caused by a massive production of superoxide anion radicals (⋅O_2_) upon cell contact (Cocheme and Murphy, 2008; Hare et al., 2011). ⋅O_2_ stimulates the generation of reactive nitrogen species or is converted into the less reactive H_2_O_2_ by superoxide dismutase (Fang, 2004; Flannagan et al., 2009). A crucial difference between the tested oxidants is the rate at which they promote the reversible oxidation of methionine residues in proteins to methionine sulfoxides (Met-SO) (Drazic and Winter, 2014). In general, all tested oxidants are able to induce Met-SO formation; however, the reaction occurs considerably faster in case of HClO compared to the less reactive H_2_O_2_ (Pattison and Davies, 2001; Peskin and Winterbourn, 2001; Drazic and Winter, 2014). Met-SO generation by chloramines appears at medium rates depending on the chloramine type (Peskin and Winterbourn, 2001). Besides methionine oxidation, the oxidative modification of cysteine residues in proteins represents a second predominant reaction of HClO with cellular components (Pattison and Davies, 2001; Gray et al., 2013a). Pattison and Davies (2001) demonstrated that at molecular ratios ≤ 4:1 of HClO and proteins, 98% of applied HClO is consumed by methionine and cysteine oxidation. In addition to the lower reactivity of H_2_O_2_, *P. aeruginosa* possesses highly efficient H_2_O_2_ removal strategies such as glutathione peroxidases and catalases (Elkins et al., 1999; Lan et al., 2010) – a fact which might also contribute to the lack of Met-SO generation or cysteine oxidation by H_2_O_2_ in contrast to HClO *in vivo* (Romsang et al., 2013). An implication of both types of protein modification in HClO signaling in bacteria has been shown for *B. Subtilis* (Palm et al., 2012) and *E. coli* (Gebendorfer et al., 2012; Drazic et al., 2013; Gray et al., 2013b), but not for *P. aeruginosa* so far. To finally answer the question whether Met-SO generation represents a key factor in the described PA3177-mediated hyperattachment phenotype of *P. aeruginosa* in response to HClO and to identify potential modified residues in regulatory proteins, further analyses such as global mass spectrometry approaches as described by Rosen et al. (2009) would be required.

Using a PA3177 overexpression strain of *P. aeruginosa* PAO1, we were able to demonstrate a strong stimulating impact of PA3177 on self-aggregation, attachment and biofilm initiation whereas motility was strongly inhibited by the DGC. Similar cellular functions have been reported for the *P. aeruginosa* DGCs such as SiaD (PA0169), RoeA (PA1107) and YfiN (PA1120) (Klebensberger et al., 2009; Malone et al., 2010; Merritt et al., 2010). As a common feature, the expression of exopolysaccharide synthesis genes was affected by all mentioned DGCs (Klebensberger et al., 2009; Malone et al., 2010; Merritt et al., 2010). In general, changes in matrix production often play a role in the enhanced bacterial biofilm formation in response to most previously mentioned antimicrobials (Bagge et al., 2004; Hoffman et al., 2005; Linares et al., 2006; Klebensberger et al., 2009; Shemesh et al., 2010; Damron et al., 2011; Kaplan, 2011; Malone et al., 2012). In *B. subtilis*, for example, stimulation of biofilm formation by ClO_2_ is associated with an enhanced matrix production mediated by the histidine kinase KinC (Shemesh et al., 2010). A similar induction of pellicle formation was also observed in *P. aeruginosa* PA14, but underlying mechanisms have not been further investigated in the respective study (Shemesh et al., 2010).

In *P. aeruginosa* biofilms, the main structural components are the polysaccharides alginate, Pel and Psl (Evans and Linker, 1973; Friedman and Kolter, 2004; Jackson et al., 2004). In contrast to alginate, which is primarily produced by mucoid strains at a later stage of biofilm development, Pel and Psl are already involved in early steps of biofilm formation like cell adhesion of *P. aeruginosa* non-mucoid variants, such as PAO1 (Wozniak et al., 2003; Overhage et al., 2005; Colvin et al., 2012; Cooley et al., 2013). In addition, flagella are important for initial cell surface attachment and biofilm formation (O’Toole and Kolter, 1998). Both flagella and exopolysaccharides are controlled by a complex regulatory network including c-di-GMP in *P. aeruginosa* (Ha and O’Toole, 2015). Using a subset of recombinant strains with mutations in genes related to flagella function (*flgK*) and exopolysaccharide synthesis (*pelF, pslA*), we were able to demonstrate a role of the Pel and Psl exopolysaccharides in the PA3177-mediated biofilm phenotype of *P. aeruginosa* PAO1, since in NaClO-treated *P. aeruginosa* deletion mutants the increase of attachment in the presence of NaClO was strongly inhibited when *pel* and/or *psl* genes were disrupted. Moreover, no increase in biofilm formation in response to a PA3177 overexpression was observed in the respective *pel* and *psl* deletion mutants.

Since PA3177 expression was also induced by contact with human macrophages, and given the fact that infected CF lungs bear an unusual high phagocytic burden and thus severely increased levels of HClO-generating MPO enzyme (Van Der Vliet et al., 2000; Downey et al., 2009), we can imagine an implication of this DGC in the development of persistent *P. aeruginosa* strains during chronic infections. Moreover, the applied HClO concentrations of 2 μg/ml are considered as physiologically relevant, since it has been demonstrated previously that 5 ×10^6^ stimulated neutrophils are able to generate 88 ± 24 nmol HClO (which corresponds to 4–5 μg HClO) within 2 h (Kalyanaraman and Sohnle, 1985) and the numbers of invading neutrophils have been estimated by approximately 1 ×10^4^/ml during airway inflammations (Thomson et al., 2010). However, we were not able to identify a role of PA3177 in virulence and survival using THP-1 cells, since the knock-out PAO1-PA3177Ω mutant strain showed no differences compared to wildtype cells.

Previously, it has been shown that a PA3177 mutation in *P. aeruginosa* strain PA14 results in altered biofilm formation, however, the authors state that these results were not consistent (Merritt et al., 2010). Furthermore, Kulasakara et al. (2006) were able to show that a PA3177 knock-out in strain PA14 did not alter virulence or biofilm phenotypes.

The observation that in addition to PA3177 gene expression of other DGCs is induced in the presence of NaClO, in particular in the PA3177 knock-out mutant, emphasizes the importance of biofilm formation as an adaptation process for *P. aeruginosa* in response to HClO stress. Further studies have to be done to clarify if one or more DGC activities are involved in counterbalancing a PA3177 knock-out. These additional effects, the potential involvement of PA3177 in pathogenesis of *P. aeruginosa* as well as the regulatory cascade involved in the regulation of PA3177 gene expression will be investigated in more detail in the future.

**FIGURE 6:**
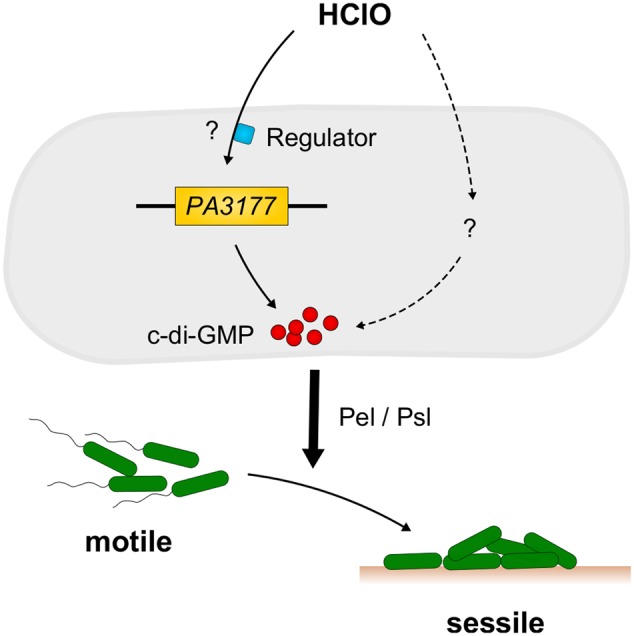
Model of HClO-induced biofilm formation in *P. aeruginosa* PAO1. Sublethal concentrations of HClO-releasing agents NaClO and Ca(ClO)_2_ are able to induce expression of *P. aeruginosa* PA3177 via a yet unknown regulatory pathway. PA3177acts as a DGC and catalyzes the formation of the second messenger c-di-GMP. These high intracellular c-di-GMP concentrations promote the switch from a free-living motile to a surface-associated sessile lifestyle and contribute to the development of persistent *P. aeruginosa* infections. Mutant analyses revealed an important role for the Pel and Psl exopolysaccharides as effectors in the PA3177-dependent pathway.

Concluding, within this work we were able to identify the host defense compound and strong disinfectant HClO as a new environmental stimulus for c-di-GMP synthesis and biofilm formation in *P. aeruginosa* (**Figure 6**). The finding that expression of the HClO-responsive DGC PA3177 is also induced upon contact of *P. aeruginosa* cells with human macrophages highlights its potential role during host–pathogen interactions.

## Author Contributions

JO and GB-W conceived the experiments. NS and AN performed susceptibility tests, biofilm, motility and phagocytosis experiments and plasmid constructions. MN performed c-di-GMP quantification by LC-MS. NS, GB-W, and JO wrote the manuscript. All authors contributed to the final version of the manuscript, and all authors approved the final manuscript.

## Conflict of Interest Statement

The authors declare that the research was conducted in the absence of any commercial or financial relationships that could be construed as a potential conflict of interest. The reviewer JR and handling Editor declared their shared affiliation.
