# Supplementary material for: The Oxidative Stress Agent Hypochlorite Stimulates c-di-GMP Synthesis and Biofilm Formation in Pseudomonas aeruginosa
Source: Front Microbiol. 2017 Nov 22;8:2311. doi: 10.3389/fmicb.2017.02311 (PMC5702645; doi:10.3389/fmicb.2017.02311)
Supplement: Supplementary file 1 [file Data_Sheet_1.pdf]

Supplementary Material

The oxidative stress agent hypochlorite stimulates c-di-GMP synthesis and biofilm formation in *Pseudomonas aeruginosa*

Nikola Strempel, Michael Nusser, Anke Neidig, Gerald Brenner-Weiss, and Joerg Overhage*

*** Correspondence:** Corresponding Author: joerg.overhage@carleton.ca

1.1 Supplementary Figures

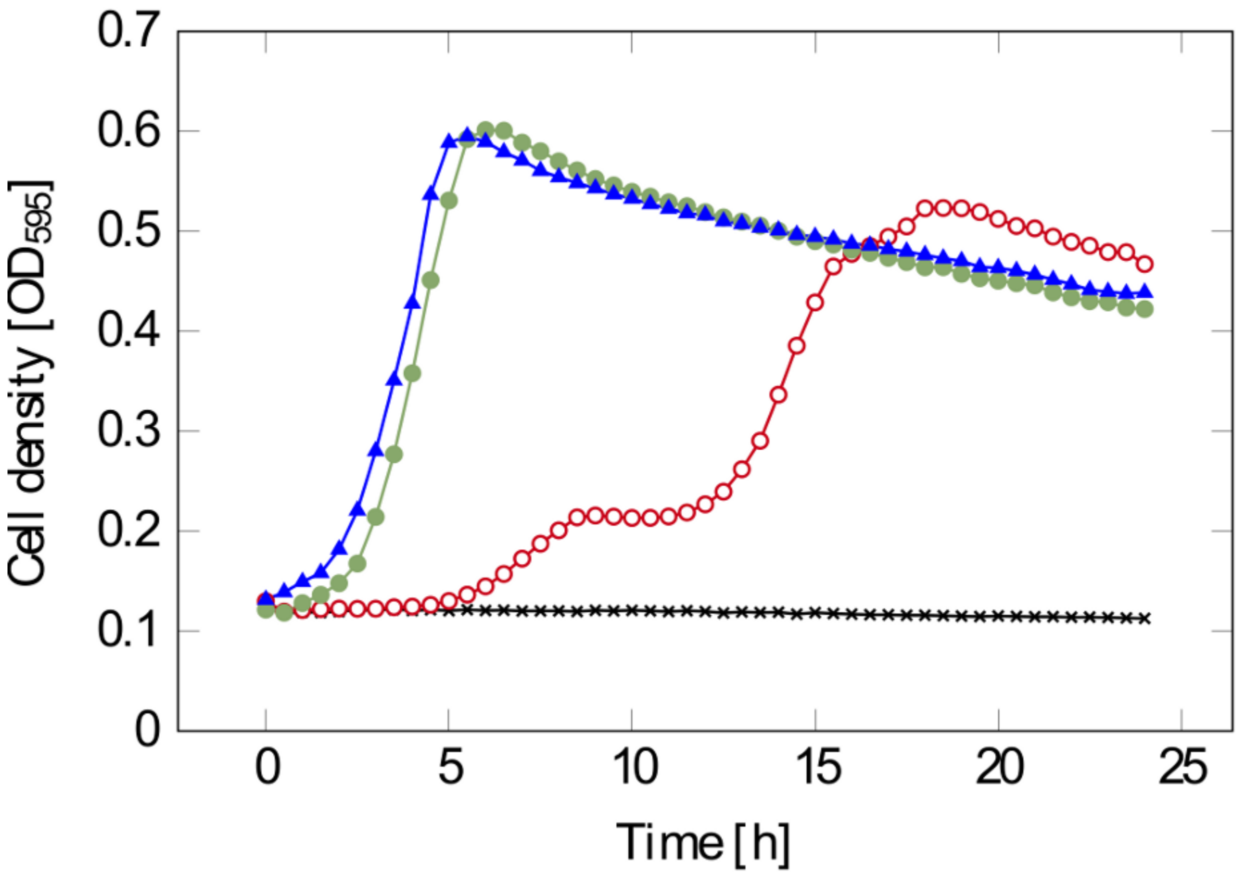

Supplementary Figure 1: Planktonic growth of *P. aeruginosa* PAO1 in the presence of NaClO.Planktonic growth of *P. aeruginosa* PAO1 at 37 °C in BM2 in the presence of NaClO was monitored over 24 h using a microtiter plate photometer at 595 nm. NaClO was added at following concentrations: 5 µg/ml (black), 4 µg/ml (red) and 2 µg/ml (green). Control cultures did not contain NaClO (blue).

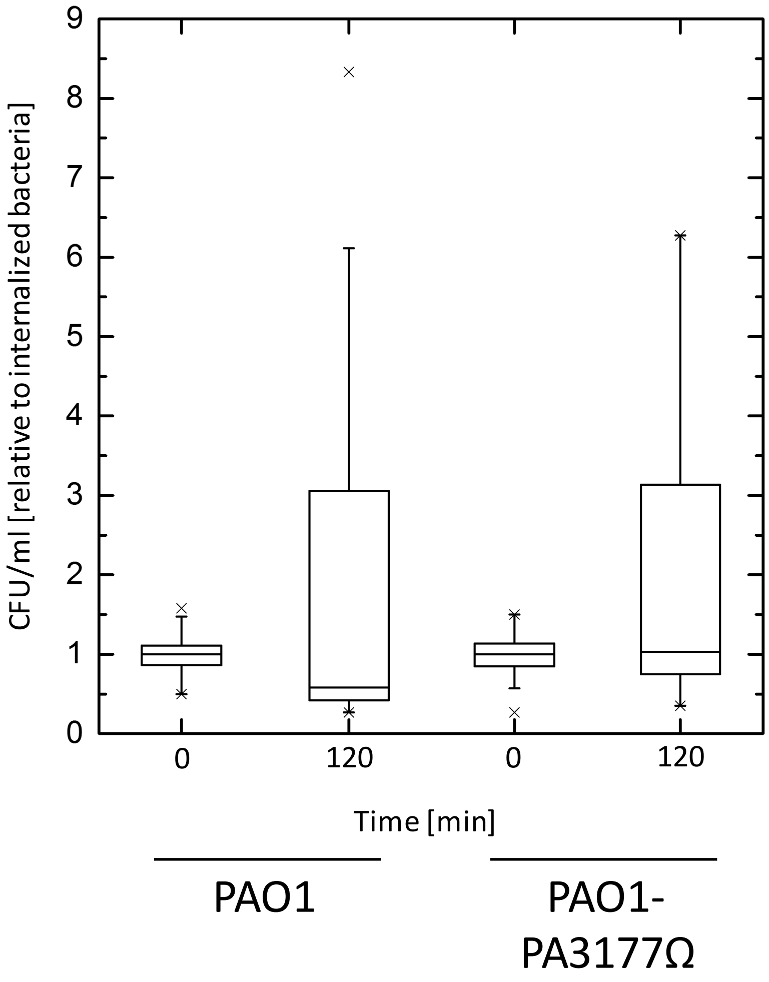

Supplementary Figure 2: Phagocytosis of *P. aeruginosa* PAO1 by THP-1. PAO1 and PAO1-3177Ω were incubated with THP-1 cells at an MOI of 10 for 1 h. Extracellular and attached bacteria were killed by treatment with gentamicin. After an additional incubation period of 120 min, infected THP-1 cells were lysed with Triton X-100, and lysates were plated to count viable intracellular bacteria. Each boxplot represents data of 3 independent biological repeats, each assayed in triplicate.

1.2 Supplementary Tables

Table S1: Cloning and sequencing oligonucleotides

Designation	DNA sequence (5'- 3')[a]	Restriction sites
PA3177_pJN105_for	AAAAATCTAGACTCGCCTTAGCCCTACTGTC	XbaI
PA3177_pJN105_rev	AAAAAGAGCTCCTTCGGGGTGTGCGAGTC	SacI
PA3177_pET23a_for	AAAAAGGATCCATGGCTCCTCCCAAGCAGT	BamHI
PA3177_pET23a_rev	AAAAACTCGAGGGCGCAGCGCAGG	XhoI
pJN105_seq_for	GGACCAAAGCCATGACAAAA	none
pJN105_seq_rev	GTGCTGCAAGGCGATTAAGT	none

[a] Restriction sites are indicated with underlined characters.

Table S2: Primers used for qRT-PCR

Target gene	Forward primer (5'- 3')	Reverse primer (5'- 3')
PA0169	GCAATGCTCGATGTGGACTTCT	ATTCGCGTAGCTCGGACTCC
PA0285	TGAGCCTGGCTTATCTCGCA	GCTTGGCGGTCATCCAGAA
PA0290	AGCAACTCTTCAGCGCCTTG	CGAAGATTTTCTCGTAGGCGG
PA0338	AGACTGCCTGCCATTCGGTAG	CGTGACAGCGGAAGAAGACCT
PA0575	CCAACGCCTTCCATGCCTA	TCGTTCACGTCGATCAGCG
PA0576	GGGGATCAACGTATTCGAGA	CAGTTCCACGGTACCCATTT
PA0847	TAGACTCATGCTGTCGCCCATT	AAGAGCCAGATACCGCCGAG
PA0861	AGGACATGCTCGAGGATCCC	TCGACGGTCTCGACGAACTC
PA1107	GCTGGAGGAGAAGAATCGCC	GCTCCATGAGCACCAACTGG
PA1120	AGCATCGCGAGCAATTGG	ATGTTGACCAGCACGGTGTC

PA1181	CCGGCCTGAGTTCCTTCAA	TGATGGAGTCGACGATGGC
PA1433	CGGCGATGGTGCAACTGAT	CGAAGTAGCCGCTGTCGAAG
PA1727	ATCTCCATCGACGACTTCGG	CGTTGATGAAGCCACGGTC
PA1851	GCTGGTCTGCCGGATAATCC	CCAGCTGAGAAACTCGCGAA
PA2072	AGCCCATGCGAAGATCCAG	CTGGAACAGCTTGTTGCGGT
PA2567	CAATGGAACGCACGGAACA	CGATGTCGTAGGTTTTGGCG
PA2771	GGAACTGACCATGATCGCCA	CGGCACTGATGGTCACTGG
PA2870	CACAACCGCCACTCGTTCCT	GCCGTGGCTGTCGTTGATGT
PA2968	GCATCCCTCGCATTCGTCT	GGCGCTCTTCAGGACCATT
PA3064	CCAGCCATACCTTCAGCCA	CTCGCGTACGAAGTCGACC
PA3177	TCGGTGCTGATGCTCGATATC	GCAGGTGGTCCTTGATGGTTT
PA3258	TTTCATTGCGACGAGAGCGT	CGTTCGGAAAGCGAGAGGAT
PA3311	GGAAAATGCCGAAACCGG	GGTGGCATTGTGCTTCCAGTAT
PA3343	AAGGACCGGTTGGACGACTC	ATGTAGCGCCGGTAGTCACG
PA3702	ATACCTGGAGATGGAGTGGCG	GCCGAAGGTGTCGTTGTAGCT
PA4332	TTCCGGCATCTACCTGGTAGAG	ACAGGCTCAGCCAGACCATTAC
PA4367	GCCCGCCCTATAGCGAATAC	GAGAATGCCGGAGATGAAGATG
PA4396	CTGGACGAAAGCATCAACCA	AGTTGCTCGTTCATTGCGG
PA4601	TCTACGAGACAGGCATCCCG	TTGAGCCCGGAGAGGATCT
PA4843	GCCTCGAACACAAGCTGCC	TGTGTCGGTTTCGCTGTCG
PA4929	GCAATTCCGACGTTTTCACTG	AAGAAGGTGGCGAAATGATGG
PA4959	CTGACCGTGCAGTTCATCAAGA	GCTTCTGCTGTTCGTGCAGTT
PA5017	TGTCGCTGTCGAAGGTCTACG	TAGGCGAGTTTCTCGATGTGCT
PA5295	AAGATCGACCGCAGCTTCG	TCGGCAACCACTTCCAGGT
PA5442	GTGGATACGCGGAAGAATGG	GGTGAAGAAACCGACGATGTG
PA5487	AGCAGGATGCCAAGGCGTT	CTTTCTGTCGCTGGTCCTCCA
